# The Prevalence and Management of Dehydration amongst Neonatal Admissions to General Paediatric Wards in Kenya—A Clinical Audit

**DOI:** 10.1093/tropej/fmx108

**Published:** 2018-01-09

**Authors:** Samuel Akech, Beatrice Rotich, Mercy Chepkirui, Philip Ayieko, Grace Irimu, Mike English, Clinical Information Network authors, Samuel N’gar N’gar, Nick Aduro, Ivan Injira, Loice Mutai, Christine Manyasi, David Kimutai, Caren Emadau, Cecilia Mutiso, Celia Muturi, Charles Nzioki, Supa Tunje, Francis Kanyingi, Agnes Mithamo, Magdalene Kuria, Sam Otido, Anne Kamunya, Alice Kariuki, Grace Wachira, Peris Njiiri, Rachel Inginia, Melab Musabi, Barnabas Kigen, Sande Charo, Grace Akech, Lydia Thuranira, Morris Ogero, Thomas Julius, Boniface Makone, Mercy Chepkirui, Wycliffe Nyachiro, James Wafula

**Affiliations:** 1 Kenya Medical Research Institute, Wellcome Trust Research Programme, Nairobi, Kenya; 2 Department of Paediatrics and Child Health, College of Health Sciences, University of Nairobi, P.O. Box 19676–00202, Nairobi, Kenya; 3 Centre for Tropical Medicine and Global Health, Nuffield Department of Medicine, Oxford University, Old Road campus, Roosevelt Drive, Headington, Oxford OX3 7FZ, UK

**Keywords:** neonates, dehydration, Kenya, Africa

## Abstract

An audit of randomly selected case records of 810 patients admitted to 13 hospitals between December 2015 and November 2016 was done. Prevalence of dehydration was 19.7% (2293 of 11 636) [95% CI: 17.1–22.6%], range across hospitals was 9.4% to 27.0%. Most cases with dehydration were clinically diagnosed (82 of 153; 53.6%), followed by excessive weight loss (54 of 153; 35.3%) and abnormal urea/electrolytes/creatinine (23 of 153; 15.0%). Documentation of fluids prescribed was poor but, where data were available, Ringers lactate (30 of 153; 19.6%) and 10% dextrose (18 of 153; 11.8%) were mostly used. Only 17 of 153 (11.1%) children had bolus fluid prescription, and Ringer’s lactate was most commonly used for bolus at a median volume per kilogram body weight of 20 ml/kg (interquartile range, 12–30 ml/kg). Neonatal dehydration is common, but current documentation may underestimate the burden. Heterogeneity in practice likely reflects the absence of guidelines that in turn reflects a lack of research informing practical treatment guidelines.

## INTRODUCTION

Neonatal dehydration may result from lactation failure associated with first-time motherhood, maternal illness, inadequate lactation support for mothers, early post-delivery discharge and incorrect use of formula feeds as well as neonatal illnesses [1–8]. It may cause peripheral gangrene, convulsions, central venous and aortic thrombosis, coma and even death in the acute period; long-term neurodevelopmental abnormalities have also been reported [9–15]. However, most studies focus on hypernatraemic dehydration, and few have studied its overall prevalence. One registry study done in the Netherlands, which included children aged up to 3 months, reported a low incidence of dehydration in infants of 58/y/100 000 breastfed infants [16]. In Kenya, clinicians within 13 hospitals that are part of the Clinical Information Network (CIN) [17] reported concerns that they frequently admit neonates who are apparently well on discharge from maternal care units with dehydration to paediatric wards. They wished to have a better estimate of the prevalence of this condition and examine its management. Neither World Health Organization (WHO) nor Kenyan guidance provides explicit recommendations for diagnosis or fluid management of neonatal dehydration [18] and practice may vary across hospitals [19, 20]. As a first step to understanding the problem of neonatal dehydration admissions to general paediatric wards, we sought to estimate its prevalence, using a broad pragmatic definition, and describe fluid management practices across the 13 hospitals.

## METHODS

### Study setting

The Kenya Medical Research Institute (KEMRI)/Wellcome Trust research programme (KWTRP), the Kenya Paediatric Association and Kenya’s Ministry of Health have been involved in a collaborative effort with hospitals since September 2013 to develop the CIN [17]. Common data on processes of care from admission clinical assessment, diagnosis, immediate treatment and discharge are collected in children aged >30 days, the age group to whom most guidelines apply. Children aged <30 days (neonates) only have minimal information collected including age, sex, weight and outcome, which are required for routine reporting. Once patients are discharged, data are retrieved from files and entered into an electronic database with de-identified data synchronized to a central server that is managed by the KWTRP. Clinical decisions on treatment are made by routine hospital staff. A detailed description of CIN including its data management processes is provided elsewhere [21, 22]. Ethical approval for CIN work and use of routine de-identified data for audit has been granted by the Scientific and Ethical Review Unit of KEMRI.

### Study objective

Our primary objective was to estimate the prevalence of dehydration in neonates admitted to paediatric wards of CIN hospitals using a broad pragmatic definition. We also aimed to describe variability in care, spanning assessment, fluid management and diagnosis across hospitals.

### Study population

We used the CIN database to identify all children aged <1 month (neonates) admitted to each of the CIN hospitals in the period December 2015 to November 2016. We then randomly selected cases for further case record review by trained data clerks at the hospitals. Data were entered into a specially designed online audit tool/database. Where a case record could not be traced, a new randomly selected case was identified.

### Sample size

We calculated sample size based on a precision of 5% around a prevalence of 50% for each hospital and a design effect of 2 for the 13 clusters (hospitals). The number obtained was inflated by 10% to account for missing medical files. Based on these assumptions, 65 randomly selected neonatal cases in each hospital were deemed sufficient to provide an acceptable estimate of the prevalence of neonatal dehydration among all neonatal admissions. We also collected data on the total number of neonatal admissions to each hospital for a year (December 2015 and November 2016).

### Case definition

Dehydration in an infant aged <1 month was defined as any of the following: a clinical diagnosis of dehydration at admission or discharge, use of a fluid bolus at admission (written in the treatment sheet or fluid chart as bolus, stat, immediately, rapidly or to run in <15 min), weight loss (current weight minus birth weight) >15% or 20% in term and preterm infants, respectively, in those aged ≤1 week, prescription of daily fluid or feed volumes >20% of requirements for a given age and body weight for children not receiving phototherapy or abnormal electrolytes (any of serum sodium >150 mmol/l, serum urea >10 mmol/l or serum creatinine >80 µmol/l if aged ≥2 days). The case definition allowed identification of neonates likely to have dehydration of some degree based on clinical, biochemical or treatment criteria, as it was believed that clinicians may fail to document dehydration as a diagnosis.

### Data analysis

We calculated the unadjusted proportion and 95% confidence interval for neonatal dehydration within the CIN network as a whole in each hospital and used inverse probability weighting to provide a final weighted overall prevalence across the 13 hospitals. Characteristics of the sampled population are described using proportions for binary, nominal or categorical data, means (±standard deviations) for normally distributed continuous data and medians (and interquartile ranges) for skewed or ordinal data. We also describe the extent of missing information for various patient assessment characteristic and treatments.

## RESULTS

**Fig. 1.**
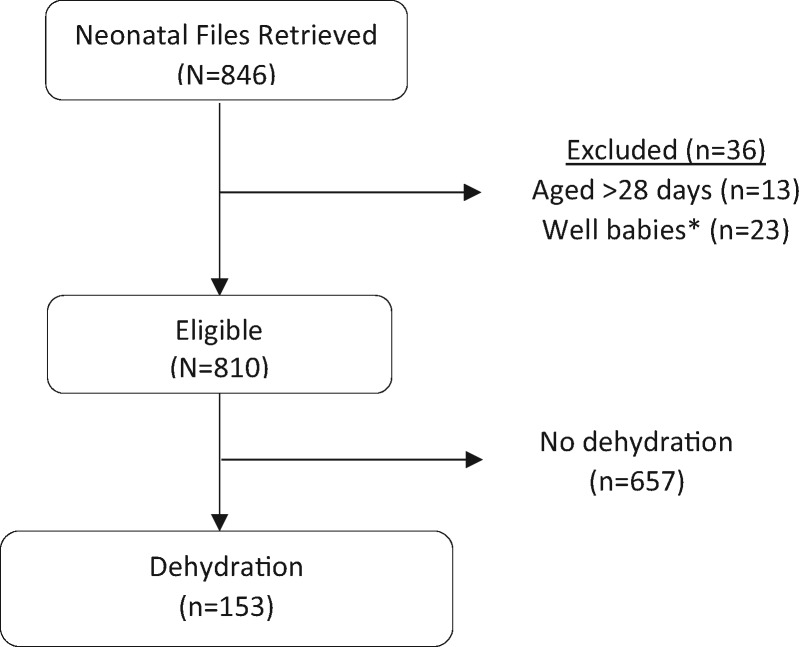
Flow diagram showing patient selection. *Well babies where the sole diagnosis was indicated as abandoned, stable baby, admitted owing to mother’s condition, normal baby, twin delivery, HIV sero-exposed or admitted for accommodation.

Randomly selected case records of 846 (100% target) patients admitted between December 2015 and November 2016 were reviewed. We excluded data from 36 patients who were >28 days (*n* = 13) or were well babies (*n* = 23); therefore, 810 (96% target) case records were eligible for evaluation for dehydration (Fig. 1) and are described in Table 1. There was poor documentation in some variables of interest, and this may have limited identification of dehydration cases. For example, APGAR score at 5 min or timing of rupture of membranes was missing in >50% of records and were not further analysed. Key information such as feeds, fluids and on any abnormalities affecting breastfeeding was missing in >80% of the records. In fact, three hospitals had no information on fluid or feed volumes at all. Despite limitation in documentation, we identified 153 cases with dehydration based on study criteria. Most cases were clinically diagnosed (82 of 153; 53.6%), followed by excessive weight loss (54 of 153; 35.3%), abnormal urea/electrolytes/creatinine (23 of 153; 15.0%) and use of extra fluid in the absence of phototherapy, which identified 2 of 153 (1.3%) cases. Only four hospitals did urea, electrolytes, and creatinine (UEC), and only 9 of 82 (11%) of those with a clinical diagnosis of dehydration had UECs done, and these were abnormal. Positive signs suggestive of dehydration were uncommon (<15% cases) in the overall population but were more common in those with dehydration.

**Table 1:** Patient characteristics

Characteristic	Overall population (*n* = 810)		Dehydration (*n* = 153)	
	Present *n*(%)	Missing *n*(%)	Present *n*(%)	Missing *n*(%)
Demographics				
Female sex	397/799 (49.7)	11 (1.4)	79/150 (52.7)	3 (2.0)
Age <1 day	69/791 (8.7)	19 (2.3)	4/151 (2.6)	2 (1.3)
Birth weight <2.5 kg	116/729 (15.9)	81 (10.0)	16/143 (11.2)	10 (6.5)
Gestation <35 weeks	62/439 (14.1)	371 (45.8)	2/79 (2.5)	74 (48.4)
Birth history				
Caesarean delivery	185/769 (24.1)	41 (5.1)	33/144 (22.9)	9 (5.9)
Home delivery	59/179 (33.0)		8/35 (22.9)	118 (77.1)
Other hospital delivery	120/471 (25.5)		27/90 (30.0)	63 (41.2)
Hospital readmission	10/689 (1.5)	121 (14.9)	3/134 (2.2)	19 (12.4)
Referred to hospital	128/678 (18.9)	132 (16.3)	21/137 (15.3)	16 (10.5)
Birth complication				
Apgar score ≤3 at 5 min	4/307 (1.3)	503 (62.1)	0/52 (0.0)	101 (66.0)
Premature rupture of membranes	111/343 (32.4)	467 (57.7)	24/72 (33.3)	81 (52.9)
Predisposing				
Abnormalities affecting breastfeeding	17/26 (65.4)	784 (96.8)	5/8 (62.5)	145 (94.8)
Can suck/breastfeed	436/751 (58.1)	59 (7.3)	63/143 (44.1)	10 (6.5)
Clinical features				
Diarrhoea	32/788 (4.1)	22 (2.7)	8/150 (5.3)	3 (2.0)
Bloody diarrhoea	1/32 (3.1)		0/8 (0.0)	145 (94.8)
Dry nappies	16/539 (3.0)	271 (33.5)	7/99 (7.1)	54 (35.3)
Delayed skin pinch	36/464 (7.8)	346 (42.7)	25/91 (27.5)	62 (40.5)
Delayed capillary refill time	19/692 (2.7)	118 (14.6)	10/131 (7.6)	22 (14.4)
Sunken anterior fontanelle	0/581 (0.0)	229 (28.3)	0/110 (0.0)	43 (28.1)
Temperature gradient	25/551 (4.5)	259 (32.0)	5/101 (5.0)	52 (34.0)
Weak pulse	38/641 (5.9)	169 (20.9)	12/127 (9.4)	26 (17.0)
Management				
Breastfeeding taught	200/433 (46.2)	377 (46.5)	34/81 (42.0)	72 (47.1)
Common primary diagnoses				
Neonatal sepsis	411/805 (51.1)		85/153 (55.6)	
Jaundice	124/805 (15.4)		16/153 (10.5)	
Birth asphyxia	43/805 (5.3)		3/153 (2.0)	
Pneumonia	42/805 (5.2)		4/153 (2.6)	
Dehydration	38/805 (4.7)		38/153 (24.8)	
Prematurity	37/805 (4.6)		2/153 (1.3)	
Respiratory distress syndrome	27/805 (3.4)		1/153 (0.7)	
Outcome				
Died	43/674 (6.4)	136 (16.8)	9 (6.8)	21 (13.7)

Table describes characteristics of all neonates surveyed and those fulfilling criteria for dehydration.

**Table 2:** Prevalence of neonatal dehydration across 13 hospitals

Hospital	Number of admissions	Number of files sampled	Number with dehydration	Prevalence of dehydration (unweighted)
H1	549	64	11	17.2 (8.9, 28.7)
H2	978	61	12	19.7 (10.5, 31.8)
H3	1024	55	8	14.5 (6.5, 26.7)
H4	266	63	14	22.2 (12.7, 34.5)
H5	1006	63	17	27.0 (16.6, 39.7)
H6	1701	65	10	15.4 (7.6, 26.5)
H7	423	64	6	9.4 (3.5, 19.3)
H8	1799	65	15	23.1 (13.5, 35.2)
H9	1183	65	14	21.5 (12.3, 33.5)
H10	980	62	11	17.7 (9.2, 29.5)
H11	1049	63	15	23.8 (14.0, 36.2)
H12	1125	56	11	19.6 (10.2, 32.4)
H13	86	64	9	14.1 (6.6, 25.0)
Weighted prevalence	11636	810	2293	19.7 (17.1, 22.6)

Prevalence varied considerably across hospitals, range 9.4%–27.0% of individual hospitals’ neonatal admissions to paediatric wards.

### Prevalence of dehydration

The overall prevalence of dehydration in the eligible population adjusted for the sample proportion from each hospital was 19.7% (2293 of 11 636, 95% confidence interval: 17.1–22.6%, with the crude proportion being 18.9%, 153 of 810). Prevalence varied considerably across hospitals, range 9.4% to 27.0% of individual hospitals’ neonatal admissions to paediatric wards (Table 2).

### Patient characteristics

In neonates with dehydration, sex distribution was similar, 22% had low birth weight (<2.5 kg), few were born at <34 weeks of gestation, clinical features of dehydration or impaired perfusion (dry nappies, delayed skin pinch, delayed capillary refill time, weak pulse) were more common than in the overall population, no participant had a sunken anterior fontanelle documented and 6.6% died in hospital. Median age was 6 days (interquartile range 3–14) and median duration of admission was 5 days (interquartile range, 2–7) in children with dehydration.

In neonates with dehydration, neonatal sepsis was the most common diagnosis present in 55.6% (85 of 153) of the cases, with dehydration recorded as a primary diagnosis in 24.8% (38 of 153), jaundice in 10.5% (16 of 153), while <5% cases had diagnoses of pneumonia, birth asphyxia or prematurity among others (Table 1). Jaundice was diagnosed slightly more often in cases with dehydration than among all neonatal admissions, while birth asphyxia, prematurity and respiratory distress syndrome were more common among the wider neonatal population than in those with dehydration. Mortality in neonates with dehydration and the broader neonatal group was similar [9 of 132 (6.8%) and 43 of 674 (6.4%), respectively].

### Fluid management of neonatal dehydration

Use of intravenous fluid was documented in 49.9% (76 of 153) of children with dehydration, but 5.9% (9 of 153) missed information on use of intravenous fluid. Feeds were documented in 20.3% (31 of 153) cases, with rest having no information on feeds. Common intravenous fluids administered in children with dehydration were Ringers lactate (30 of 76; 39.5%) and 10% dextrose (18 of 76; 23.7%). Other fluids including half-strength Darrow’s, oral rehydration solution, normal saline, 5% dextrose/half-strength Darrow’s mix and Ringer’s lactate/10% dextrose mix were administered in six or fewer children. Eighteen children (11.7%) were managed with expressed breast milk or milk alone without intravenous fluids or oral rehydration solution. The median volume per kilogram body weight administered was 80 ml/kg (interquartile range 75–170 ml), and the median duration of fluid administration was 6 h (interquartile range 4–24 h). Seventeen children received a bolus of fluid, and 16 of them had information on the fluid type used. Ringer’s lactate was most commonly used (63%; 10 of 16), 5% dextrose water and Ringer’s lactate/10% dextrose mix were given to one patient each, while four other patients received a variety of fluids. Majority, 15 of 17 (88%), of bolus recipients only got one bolus of median volume 20 ml/kg (interquartile range 12–30 ml/kg. Bolus was mostly given rapidly, 9 of 17 (52.9%), over 1–2 h in 5 of 17 (29.4) cases and with no information on two cases.

## DISCUSSION

This clinical audit was intended to investigate the prevalence and management of neonatal dehydration in neonates admitted to paediatric wards of 13 hospitals in Kenya. While these hospitals were not selected to be a representative sample of all hospitals in Kenya [17], they do provide a much broader picture of routine care than is available from most countries in sub-Saharan Africa. The audit was prompted by clinicians concerned that neonatal dehydration is common and that care is not supported by effective guidelines. Findings suggest one-fifth of neonates admitted to these hospitals’ paediatric wards have dehydration. The majority were identified based on a clinical diagnosis, while abnormal renal function and excessive weight collectively identified about 45% of the cases. We failed to find comparable studies to compare the prevalence of neonatal dehydration. We did find a study conducted in the Netherlands, which found a low prevalence, that used registry data but relied on reported information, and underreporting may be problem in such cases [16].

The criteria for diagnosis of dehydration were broad, including evidence from fluid prescriptions, and may inflate the estimate of prevalence. However, documentation was often poor and access to renal function testing very limited. In addition, a number of studies report that weight loss, even <10% [3, 23–25], is a sensitive marker of neonatal dehydration, but we used a higher cut-off for weight loss. Overall, this may therefore result in underestimation of prevalence. We also noted that prevalence of dehydration varied across hospitals (4.8%–25.4%), and while differences in documentation may explain this, it may also be a result of differences in underlying case mix.

Poor documentation of treatment limited detailed description of fluid management, but the limited data suggest variation in fluids used, variation in use of boluses, variation in volumes prescribed and that volumes used may be insufficient to treat dehydration. Dextrose 10% with electrolytes and Ringer’s lactate, which were the commonly prescribed fluids, are commonly used as maintenance fluids in neonates or for treating dehydration in older infants, respectively (WHO 2005, 2013). Weight loss is often accompanied by hypernatraemia [23, 25, 26], which should be corrected gradually during dehydration, as rapid reduction of serum sodium may result in rapid shifts of water between fluid compartments, leading to cerebral oedema, pontine myelinosis, coma and even death [27–29]. Therefore, widespread lack of electrolyte testing in these hospitals both at the point of diagnosis and during treatment should be of concern.

## CONCLUSION

Neonatal dehydration is common in neonates admitted to paediatric wards across first-level referral hospitals in Kenya, but current documentation and supportive laboratory testing may underestimate the burden. Documentation of fluid management is poor, but data suggest wide variation in choice of fluid, rate and volumes administered. Defining best practice in this condition would be useful, and researchers need to address the current gaps in evidence to inform future guidance.
